# Fever of unknown origin associated with immune checkpoint inhibitors

**DOI:** 10.3389/fimmu.2024.1364128

**Published:** 2024-03-12

**Authors:** Xu Tong, Tao Zhan, Xiaoqin Dong, Dong Xu

**Affiliations:** ^1^ The Second Clinical Medical College, Tongji Medical College, Huazhong University of Science and Technology, Wuhan, Hubei, China; ^2^ Department and Institute of Infectious Disease, Tongji Hospital, Tongji Medical College and State Key Laboratory for Diagnosis and Treatment of Severe Zoonotic Infectious Disease, Huazhong University of Science and Technology, Wuhan, Hubei, China

**Keywords:** immune checkpoint inhibitors, fever, cytokines, FUO, irAEs

## Abstract

Since the approval for the treatment of melanoma in 2014, immune checkpoint inhibitors (ICIs) have revolutionized the therapy pattern across various malignancies. Coinciding with their frequent usage, their adverse effects, including fever, cannot be neglected. In the context of cancer diseases and cancer treatments, fever of unknown origin (FUO), which has long posed a challenge for clinicians in terms of diagnosis and management, brings forth new connotation and significance. In this paper review, we present the concept of ICIs-associated FUO, consider activated immune system and elevated cytokines as common mechanisms by which ICIs induce fever and various immune-related adverse events (irAEs), summarize and compare the primary etiologies of ICI-associated FUO, and compare it with conventional types of FUO.

## 1 Introduction

Cancer cells attenuate the anti-tumor immune response, promote their proliferation and metastasis through the expression of immune checkpoint proteins (1). Immune-checkpoint inhibitors (ICIs), which reverse aforementioned tumor-mediated immune evasion by blocking immune checkpoints, have revolutionized the treatment of malignant tumors since the approval in 2014 (2). Currently, FDA-approved ICIs comprise monoclonal antibodies targeting the PD-1 - PD-L1 axis or CTLA-4 - CD28 axis (3, 4) (**Table 1**). Concomitant with the surge of ICIs usage, immune-related adverse events (irAEs) have garnered increasing attention. The irAEs involve respiratory, digestive, nervous, hematological, and endocrine systems (5), and imbalances in their immune status may be accompanied by fever.

**Table 1:** Immune checkpoint inhibitors approved by the Food and Drug Administration.

ICIs	Target	Clinical applications	Trade name^®^	Date of approval
Cemiplimab	PD-1	BCC, CSCC, NSCLC	Libtayo^®^	Sep, 2018
Dostarlimab	PD-1	Endometrial carcinoma	Jemperli^®^	Aug, 2021
Nivolumab	PD-1	CRC, ESCC, HCC, M, NSCLC, RCC, UC, etc	Opdivo^®^	Dec, 2014
Pembrolizumab	PD-1	Breast cancer, CRC, EC, GC, HCC, M, NSCLC, RCC, SCLC, UC, etc	Keytruda^®^	Sep, 2014
Atezolizumab	PD-L1	Breast cancer, HCC, M, NSCLC, SCLC, UC	Tecentriq^®^	May, 2016
Avelumab	PD-L1	MCC, RCC, UC	Bavencio^®^	May, 2017
Durvalumab	PD-L1	NSCLC, SCLC, UC	Imfinzi^®^	May, 2017
Ipilimumab	CTLA-4	CRC, HCC, M, NSCLC, RCC	Yervoy^®^	Mar, 2011
Tremelimumab	CTLA-4	HCC	Imjudo^®^	Oct, 2022
Relatlimab plus Nivolumab	LAG-3 plus PD-1	M	Opdualag^®^	Mar, 2022

BCC, basal cell carcinoma; CRC, colorectal cancer; CSCC, cutaneous squamous cell carcinoma; ESCC, esophageal squamous cell carcinoma; GC, gastric carcinoma; HCC, hepatocellular carcinoma; M, melanoma; MCC, merkel cell carcinoma; NSCLC, non-small cell lung cancer; RCC, renal cell carcinoma; SCLC, small cell lung cancer; UC, urothelial carcinoma.

For over a century, fever of unknown origin (FUO) has been a diagnostic and therapeutic challenge for clinicians. FUO, characterized by the inability to clarify the cause of fever despite reasonable investigations in inpatient or outpatient settings, lacks consensus regarding specific febrile temperatures or fever duration (6). In general, FUO are classified as classic FUO, nosocomial FUO, immunodeficiency-associated FUO, and travel-associated FUO. Given that 1) ICIs are administered more frequently in cancer therapy; 2) pyrexia stands as one of the most common adverse events of ICIs (7, 8); 3) patients treated with ICIs face challenges in identifying and discriminating the etiology of FUO, we believe that it is of crucial clinical significance to list ICIs-associated FUO as a separate category of FUO, which is defined as fever during ICIs administration in cancer patients with immune activation, cytokines secretion or pathogen reactivation.

In this review, we describe the mechanisms by which ICIs leads to fever, summarize the main etiologies, pathogenesis and characteristics of ICIs-associated FUO and make comparison among them, and compare ICIs-associated FUO with conventional types.

## 2 Mechanisms of post-treatment pyrexia associated with ICIs

### 2.1 Endogenous pyrogens and other fever-related cytokines

Fever is caused by the activation of pyrogenic agents, which activate endogenous pyrogen (EP)-producing cells to generate and release EP. This results in an upward adjustment of the body temperature set point (SP), leading to regulated temperature elevation (6). EP mainly consists of cytokines such as interleukin (IL), tumor necrosis factor (TNF) and interferon (IFN), and the levels of these cytokines usually alter after immunotherapy (9, 10). IL-1 and IL-6 are considered to be the most important EP (9, 11), whose production and release lead to the release of prostaglandin E (PGE) from brain endothelial cells, which serves as a positive regulatory mediator of fever (6). Research has shown that blocking PGE production or attenuating its function can mitigate fever symptoms (12–14), which lays a theoretic foundation for aspirin and ibuprofen.

IFN was first found to induce fever in clinical trials, and subsequent studies have demonstrated that it entailed fever not due to contamination with endotoxins or its impact on the IL-1 pathway (15), but rather through its direct action on the thermoregulatory center. As a pro-inflammatory cytokine, TNF can result in fever through RANKL/RANK-COX2-PEG2-EP3R pathway and other experiments have proved that it can also stimulate IL-1 production both *in vivo* and *in vitro* (15, 16).

IL-2 and IL-8 have also been viewed as cytokines highly associated with fever, although there still remains controversy regarding whether they are EP in the strict sense. It has been suggested that IL-2 does not stimulate thermoregulatory center directly, but induces fever indirectly by eliciting other EP, such as circulating TNF (17). IL-8 is also thought to be associated with fever and can be utilized as a biomarker for pyrexia, infection, septicemia and neutropenia in cancer patients (17).

### 2.2 Alteration in cytokines’ level following ICIs treatment

Both PD-1/PD-L1 inhibitors and CTLA-4 inhibitors contribute to upregulation in the production and release of pro-inflammatory cytokines and even leads to the occurrence of cytokine release syndrome (CRS) in severe cases (18, 19). For instance, CTLA-4 monoclonal antibody leads to the release of TNF, IFN-γ and IL-2, which in turn further promotes T cell proliferation and activation (2). Experiments *in vitro* have demonstrated that CTLA-4 monoclonal antibody administered in melanoma cell lines will promote TNF-α production from NK cells (2). ICIs accelerate the production of IFN-γ, which increases tumor immunogenicity, curbs the proliferation and infiltration of cancer cells, attracts immune cells to malignant disease sites, and enhances the cytotoxic function of NK cells and CTLs (20). It has been demonstrated that PD-1/PD-L1 inhibitors can promote tumor-associated macrophages (TAMs) to produce IL-6, and IL-6 inhibitors can elevate Th1 responses and defers melanoma progression (21). As immune checkpoint molecules including PD-1 - PD-L1 lead to a decrease in IL-2 release, ICIs may increase IL-2 secretion to augment immune response (22). To the best of our knowledge, it is commonplace for patients to experience elevated levels of certain cytokines after ICIs treatment (**Table 2**), although the mechanisms of each cytokine’s alteration has not been thoroughly investigated and elucidated (20, 31).

**Table 2:** Elevation in cytokines’ level following ICIs administration.

ICIs	Tumors	N=	Elevated cytokines (of participants/%)	References
Ipilimumab	Bladder cancer	4	IFN-γ (100%)	(23)
PD-1 + CTLA-4	CC	*	IFN-γ	(24)
Pembrolizumab or nivolumab	NSCLC	26	IL-1, IL-2, IL-8, TNF-α, IFN-γ	(25)
Pembrolizumab or nivolumab	NSCLC	10	IL-6 (70%), TNF-α (60%)	(26)
Nivolumab	NSCLC	27	IL-8 (85.7% of initial responding patients)	(27)
Nivolumab	M	16	IL-6 (50%)	(21)
Pembrolizumab or nivolumab	M	29	IL-8 (48.3%)	(28)
Ipilimumab + chemotherapy	SCLC	37	IL-1, IL-2, IL-6, IL-8, TNF-α, IFN-γ	(29)
PD-1 + CTLA-4	No limit	12	IL-1, IFN-γ	(30)

CC, colon cancer; M, melanoma; NSCLC, non-small cell lung cancer; SCLC, small cell lung cancer.

*mouse model.

**Figure 1:**
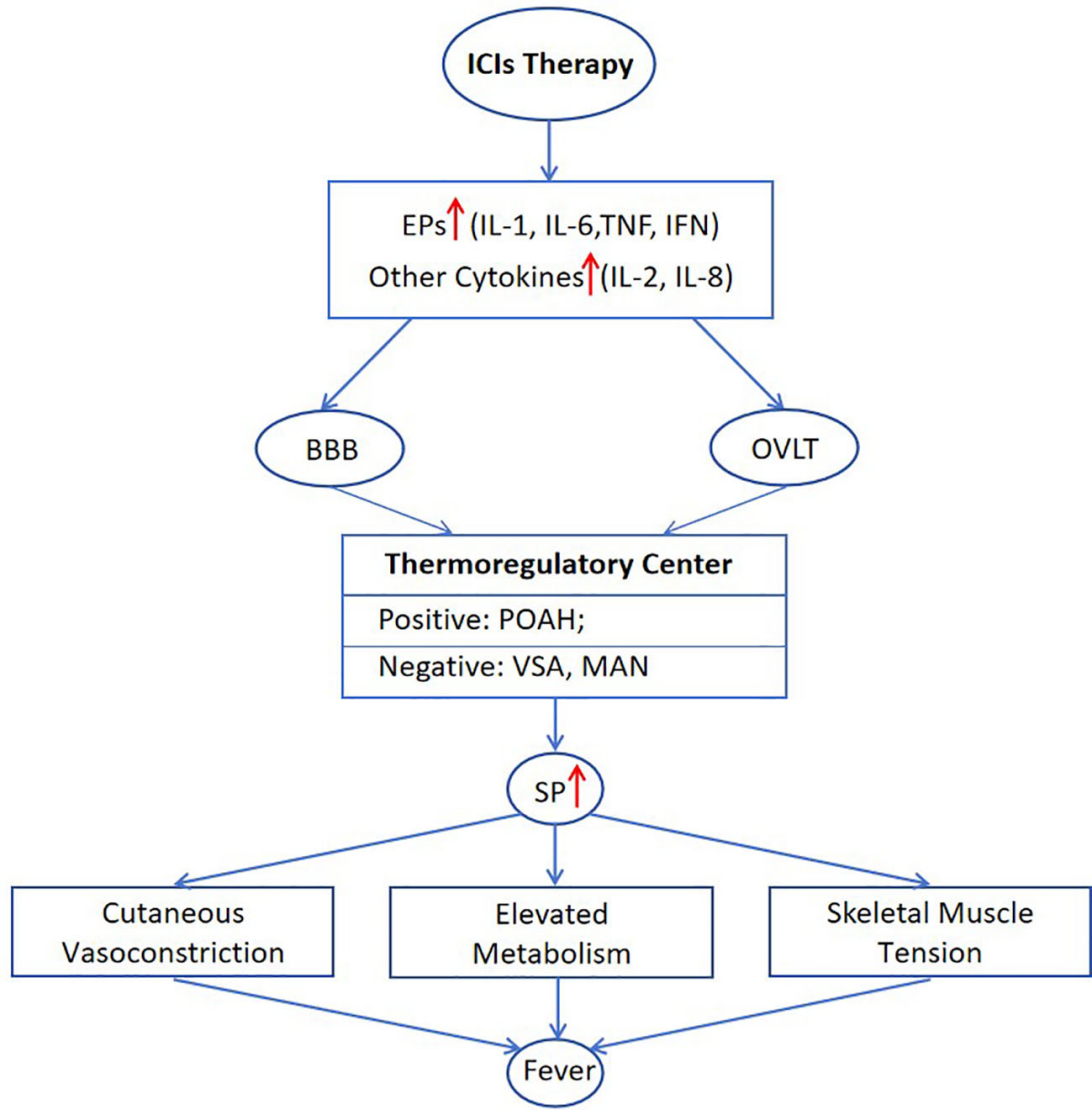
Mechanisms of post-treatment pyrexia associated with ICIs. BBB, blood-brain barrier; MAN, medial amygdaloid nucleus; OVLT, organum vasculosum laminae terminalis; POAH, preoptic anterior hypothalamus; SP, set point; VSA, ventral septal area.

To summarize, levels of IL-1, IL-2, IL-6, IL-8, TNF, and IFN in patients may undergo elevation after ICIs treatment. And these cytokines can function as EP or febrile activators, acting on the preoptic anterior hypothalamus (POAH) through blood-brain barrier (BBB) or organum vasculosum laminae terminalis (OVLT) (32). Consequently, the SP rises due to the interaction between the positive thermoregulatory center POAH and negative thermoregulatory centers including ventral septal area (VSA) and medial amygdaloid nucleus (MAN) (33–36). This eventually leads to the feverish state characterized by reduced heat dissipation and increased heat production. The possible mechanism of fever induced by ICIs therapy is illustrated in (**Figure 1**).

## 3 Etiology of fever of unknown origin induced by ICIs

Fever, caused by a wide variety of reasons, is frequently reported in cancer patients (37), and fever is one of the most prevalent adverse events after ICIs remedy (7, 8). When immunotherapy is combined with other therapy patterns, the incidence of fever increases remarkably. For example, the rate of febrile events lifts in the combination of PD-1/PD-L1 inhibitors and chemotherapy, compared with chemotherapy alone, as shown in a meta-analysis with rates of 18.8% (85 out of 452) and 10.7% (47 out of 438) respectively (37). In NSCLC, the incidence of fever is as high as 32.5% - 38.1% in patients receiving ICIs plus chemotherapy or radiotherapy (38).

Theoretically, adverse events following ICIs treatment involve nearly every system. Considering their incidence, severity, and their association with immune dysregulation and fever, we focus on the following complications, including pneumonitis, tuberculosis (TB), colitis, hepatitis, and hematological irAEs (Haem irAEs) (5). Therefore, we aim to review fever of unknown origin associated with ICIs according to above etiologies and compare their differences.

### 3.1 Pneumonitis

The prevalence of checkpoint inhibitor pneumonitis (CIP) ranges from 3.5% - 19% (39), with a mortality rate of 10% - 17% (40). The potential mechanisms by which immunotherapy leads to pneumonitis have been summarized in the following four points (41). Firstly, ICIs lead to disorder in the quantity of T cell subsets, manifested as a significant increase in CD4+ T cells, with Th1 cell infiltration playing a predominant role. There is also an increase in the infiltration of CD8+ T cells, particularly those expressing PD-1, TIM-3, and TIGIT. The reduced number and inhibited function of Tregs can also contribute to CIP. Secondly, elevated levels of autoantibodies, including rheumatoid factor (RF), antinuclear antibody, antithyroglobulin, antithyroid peroxidase and CD74 autoantibodies, also contribute to CIP (40). Thirdly, consistent with what we mentioned earlier, the increase in various inflammatory cytokines such as IL-1, IL-2, IL-13, G-CSF, GM-CSF is closely associated with high-grade irAEs. In terms of CIP, the imbalance in C-reactive protein (CRP), IL-6 and IL-17 is closely associated with disease development. Fourthly, different therapy modalities exert enormous impact. Anti-PD-1 therapy has a higher incidence rate than anti-PD-L1 and anti-CTLA-4 therapy, and combination with chemotherapy or radiotherapy raises the incidence through various mechanisms (41, 42). Additionally, intestinal flora, non-coding RNA and other immune cells (B cells, NK, DC) are also relevant to the development of CIP.

The clinical presentation of CIP mainly includes dyspnea, decreased activity tolerance, and cough, whereas fever (12%) and chest pain (7%) are less common (42, 43). Consequently, case reports of CIP reveal that fever is not the essential factor for the diagnosis of CIP. In patients who claimed pyrexia, the onset of fever occurred between week 2 and week 32 after immunotherapy, with peak temperatures ranging from 38.1°C to 40 °C (43–48). However, the fever type and daily variation of body temperature were not thoroughly documented. Organizing pneumonia (OP) is the main radiological manifestation of CIP, and bronchoscopy and bronchoalveolar lavage fluid (BALF) can be utilized for differential diagnosis (39). Biomarkers including IL-17A and IL-35 from BALF may be associated with the occurrence and severity of CIP (39).

### 3.2 Tuberculosis

Patients undergoing ICIs administration are eight times more likely to contract TB than the general population, according to an observational study (49). A Meta-analysis revealed that patients receiving PD-1/PD-L1 in developed Asian countries were 35 times more likely to develop TB than general population (50). Hence several reports have suggested that TB screening should be performed prior to ICIs treatment (51, 52). PD-1 and PD-L1 inhibitors are associated with TB reactivation, whereas CTLA-4 inhibitors appeared to have no impact (52, 53). Immune checkpoints are closely related to TB infection, as their expression is upregulated when TB is active and is downregulated when TB is curbed. Defects in PD-1/PD-L1 exacerbate TB infection, which is associated with increased infiltration of inflammatory cells and cytokines. Thus, the expression of immune checkpoints may be a necessary condition for maintaining lymphocyte function as protective responses against TB infection (54).

Upon infection with mycobacterium tuberculosis (MTB), body’s intrinsic immune cells, mainly macrophages, recognize TB through pattern recognition receptor (PRR). Cytokines including IL-1, IL-6, TNF-α will be secreted by macrophages to guide the accumulation of lymphocytes and monocytes at the location of MTB. Among them, CD4+ Th1 T cells release loads of cytokines, encompassing TNF-α, IFN-γ, IL-2, etc., gradually forming tuberculous granuloma. After that, the body represents a state of latent infection, during which PD-1 expression is elevated, inducing T cells’ apoptosis. Nevertheless, ICIs block PD-1/PD-L1, restoring lymphocytes’ function and releasing inflammatory cytokines such as IFN-γ and TNF-α. Excessive inflammatory cells and cytokines destroy the extracellular matrix, favoring the growth of MTB, which breaks the state of latent infection and leads to the reactivation of TB (53).

TB infection following ICIs therapy lacks specificity, and is usually characterized by cough, expectoration, breathlessness, fever and weight loss (49, 53). Imaging manifestations, from chest X-ray and contrast CT, support the diagnosis with features like new consolidation, patchy opacity/nodules, or the tree-in-bud sign, especially in the upper lobes (49). However, confirmation is achieved by culture of MTB or DNA detection by polymerase chain reaction (PCR) (52, 55). Besides, interferon-gamma release assay (IGRA) testing is recommended to assess the risk of developing active TB for relevant patients (56). In case reports, we find many patients are diagnosed with TB despite the absence of symptoms of shortness of breath, cough, fever, night sweats (55, 57). In case reports with detailed fever data, we found that the fever of TB infection following ICIs therapy occurred between week 13 and week 87, with peak temperatures between 38.6°C and 39.2°C (58–61).

### 3.3 Hepatitis

The incidence rate of developing immune-mediated hepatitis (IMH) in patients receiving immunotherapy ranges from 5% to 10% (62). The risk of liver toxicity is higher with CTLA-4 inhibitors, reaching up to 15%, and the figure is even higher when multiple ICIs are used in combination (63). Possible mechanisms include cytotoxicity due to complement activation, but such theory fails to elaborate why the liver would be a specific target (62). It has been proposed that ICIs block immunosuppressive signals and stimulate T cells’ function, activation and proliferation. Activated T cells, under the influence of adhesion molecules, adhere to the hepatic sinusoids. Fas receptors on activated T cells bind to FasL expressed on liver sinusoidal endothelial cells and Kupffer cells, resulting in apoptosis of activated T cells. Kupffer cells, activated by Fas/FasL binding and IFN-γ secreted by T cells, release TNF-α to render hepatocytes more sensitive to IFN-γ-mediated apoptosis, resulting in apoptosis and injury of hepatocytes ultimately (62).

Although most IMH cases are asymptomatic, a small fraction of them may exhibit fatigue (17.1%), abdominal discomfort (14.0%), fever (14.0%), rash (4.3%), and jaundice (3.7%) (64, 65). Reports indicate that fever is more prevalent in CTLA-4 inhibitors treatment than PD-1/PD-L1 inhibitors treatment (66). Elevated ALT or AST exceeding twice the upper limit of normal is regarded as an indicator of IMH (64). Imaging findings manifest hepatomegaly, peri-portal edema and lymphadenopathy, which are non-specific (62). The histological pattern of PD1/PD-L1-associated IMH tend to exhibit lobular hepatitis, whereas CTLA-4-associated IMH is more inclined to granulomatous hepatitis (66). Fever of IMH typically appears between day 3 and week 26 and ranges 38°C to 40°C (66, 67).

### 3.4 Colitis

The incidence of immune-mediated colitis (IMC) is approximately 3.6% (68). Statistics indicate that the incidence of all-grade colitis after PD-1/PD-L1 treatment is around 1% - 1.6% while the figure for CTLA-4 treatment can be as high as 8.8% - 9.1% (68). The inflammation in the colonic mucosa is attributed to higher level of cytokines released by CD4+ T cells and the mucosal infiltration of CD8+ T cells with enhanced cytotoxicity and proliferative state (69). IL-1, IL-10, IL-17 and transforming growth factor-β1 (TGF-β1) may serve as biomarkers of IMC (68).

Symptoms of IMC include abdominal pain (20%), abdominal bloating, nausea and vomiting (15%), fever (12%), along with the presence of mucus and blood in stool, and signs of peritoneal irritation (68, 70). Endoscopy is considered the gold standard for diagnosing IMC. Laboratory tests, including complete blood count, CRP and erythrocyte sedimentation rate, stool testing for infectious pathogens, and virus or parasites infections are used as adjuncts in the diagnostic process (68). Pathological biopsy lacks specificity due to its diverse histologic manifestations, but CT can evaluate the degree of inflammatory changes in IMC, which includes bowel wall thickening, mesenteric engorgement, fat stranding, and fluid-filled bowel (68). It has been observed that fever onset occurs relatively early, ranging from day 5 to week 9, with reports extending to week 43 or week 147. The peak temperature typically ranges from 37.2°C to 39°C (70–77).

### 3.5 Neutropenia

Patients receiving ICIs treatment exhibit reduced risk of neutropenia compared to those undergoing chemotherapy. Despite this, neutropenia remains one of the most common Haem irAEs, accounting for approximately one-fourth of hematologic complications (78, 79). Notably, neutropenia induced by ICIs, although rare, mostly presents as grade 4 (absolute neutrophil count < 500 cells/µL), posing a risk of fatal septic shock and is thus described as a life-threatening side effect (80, 81). In such cases, a substantial number of patients must permanently discontinue ICIs treatment (81, 82). A retrospective study based on FAERS database highlighted the incidence and severity of neutropenia, identifying fever and neutropenia as common fatal adverse events in PD-L1 monotherapy and the most prevalent fatal adverse events in PD-L1 combined with bevacizumab (83). The study highlights that combination therapies, the current research hot spot, can help patients delay tumor progression and reduce mortality compared with monotherapy (84). However, it also comes with a significantly increased incidence of neutropenia and fever. Therefore, a deeper understanding of ICIs-induced neutropenia could assist clinicians in more effectively managing irAEs and making informed treatment decisions. The mechanism underlying ICIs-induced neutropenia remains to be explored. According to the current research, we summarize that a portion of neutropenia may occur due to the activation of T cells, leading to widespread infiltration of cytotoxic T cells into the bone marrow tissue. This type is also referred to as the central type. Correspondingly, the peripheral type may arise due to the production of anti-neutrophil antibodies, impeding granulocytes’ maturation (85, 86). Therefore, immunological examination of anti-neutrophil antibodies and bone marrow examination can help identify potential causes.

In cases of ICIs-induced neutropenia, patients may not exhibit any symptoms, with abnormalities often detected through laboratory tests. Due to the role of neutrophils in innate immune defense, patients are susceptible to infection-related fever, and the proportion of febrile neutropenia typically usually exceeds 50% (85, 87, 88). While most fevers observed in ICIs-related trials are categorized as grade 1 or 2 adverse reactions, febrile neutropenia is consistently described as grade 3-5, which is most likely to result in the discontinuation of ICIs therapy (37). In a retrospective study involving 35 patients with Haem irAEs registered in three pharmacovigilance databases, 9 patients developed neutropenia, of which 6 patients (66.7%) progressed to febrile neutropenia due to infection. Notably, 2 deaths associated with ICIs were both attributed to febrile neutropenia (78). Despite the effectiveness of steroids in alleviating Haem irAEs, it is recommended to administer G-CSF and antibiotics to patients with febrile neutropenia instead of choosing systemic steroid treatment. The onset of febrile neutropenia commonly occurs in the third or fourth cycle of treatment, typically around 6 to 12 weeks after the initial ICIs treatment. The peak temperature of patients generally exceeds 38°C, with most cases ranging between 38°C and 39°C (89–96).

### 3.6 Cytokine release syndrome

Cytokine release syndrome (CRS) is relatively rare in patients treated with ICIs, as evidenced by an incidence of approximately 4.6% reported in a single-center retrospective study (19, 97, 98). However, once it occurs, it tends to be persistently severe and potentially life-threatening (99, 100). CRS, as a systemic inflammatory response mediated by inflammatory cytokines, is directly triggered by immunotherapy. It was initially used to describe the toxic side effects associated with the anti-T-cell antibody OKT3. Subsequently, it has been observed in various immune-related therapies, including anti-thymocyte globulin (ATG), rituximab, chimeric antigen receptor (CAR) T cell therapy (101–103). Thus, with the greater success of immunotherapy, there is growing interest in investigating the mechanisms, diagnosis, and treatment of CRS. T cells activated during immunotherapy can produce inflammatory cytokines such as IFN-γ, TNF, and GM-CSF. Furthermore, the lysis of tumor cells is also attributed to the elevated level of these cytokines, which can activate macrophages to secrete inflammatory mediators, including the crucial cytokines IL-6 and IL-1 (104, 105). IL-6 has been proven to actively participate in the systemic pathological response in CRS patients. It initiates the subsequent release of cytokines by signaling to non-immune tissues such as endothelial cells, triggering cascade reaction that results in severe symptoms, including vascular leakage, circulation failure, complement activation, and disseminated intravascular coagulation (106). In addition, IL-1 can also contribute to downstream cascade reaction, resulting in the release of large amounts of cytokines. Moreover, IL-1 can signal to the hypothalamus and pituitary gland to induce fever and has been found a strong association with neurotoxicity (104, 107). Given the critical role of IL-6 and IL-1 in CRS, the corresponding IL-6R inhibitors (Tocilizumab) and IL-1R inhibitors (Anakinra) have been identified for their effectiveness in treatment (19, 108, 109).

The clinical symptoms and severity of CRS exhibit wide variation. Mild cases may manifest with symptoms such as fever, headache, myalgia, and nausea, while severe cases can present with hypotension, cardiac dysfunction, cerebral edema, myocarditis, hepatic and renal failure, and other organ-related severe symptoms (19, 110). Due to the nonspecific feature of CRS symptoms, there may be overlapping symptoms with tumor lysis syndrome, infections, and neutropenia, which need to be carefully identified and considered for attribution (110). Fever, a hallmark of CRS, typically occurs around 11.0 days after the initiation of ICIs. Interestingly, patients with severe CRS may present with fever significantly later (111). CRS patients generally experience high fever, and a review of published case reports indicates that peak temperatures induced by ICIs range from 38.5°C to 40.3°C, with most cases exceeding 39°C (99, 100, 109, 112–114).

### 3.7 Hemophagocytic lymphohistiocytosis

Hemophagocytic lymphohistiocytosis (HLH) is a rare but critical immune complication characterized by an overactive immune system that results in multi-system damage (115–117). The observational study of the VigiBase database reveals an incidence of ICIs associated with HLH at approximately 0.08% (118). However, the incidence of life-threatening or fatal conditions in these HLH patients alarmingly reaches 44% (118). Similar to CRS, HLH is attributed to a systemic inflammatory response. The key pathophysiological process involves the over-activation of CD8+ T-cells and macrophages, leading to the secretion of substantial amounts of pro-inflammatory cytokines, including IFN-γ, TNF-α, IL-1, IL-4, IL-6, and IL-8. The excessive cytokine levels in serum lead to organ damage and systemic organ failure (119, 120). Current research highlights IFN-γ as a pivotal player in HLH. This inflammatory factor not only induces fever and activates macrophages but also hampers bone marrow and blood cell production, leading to cytopenia and lymph node disease (121, 122). Consequently, targeting IFN-γ emerges as a potential strategy to improve the prognosis of HLH patients.

According to the HLH-2004 and Hscore diagnostic criteria, common symptoms in patients with HLH include fever and splenomegaly (123, 124). Typical laboratory findings include 2 or 3 lineages of peripheral blood cytopenias, hypertriglyceridemia, hypofibrinogenemia, elevated serum ferritin, and sCD25 (125). The biopsy can reveal hemophagocytosis in the bone marrow, spleen, or lymph nodes (126). It is worth noting that many symptoms overlap between CRS and HLH. Severe CRS may present similar clinical and laboratory manifestations of HLH, including elevated serum ferritin and triglycerides, which makes it challenging to distinguish from primary HLH (127). Despite theoretical differences in cytokine levels, such as the higher levels of IL-6 in CRS patients, the challenge lies in the variability of baseline inflammatory cytokine levels in cancer patients, making it unreliable as a discriminatory factor (110). It has been suggested in the literature that CRP, an IL-6 reactant produced by the liver, can serve as a reliable surrogate for IL-6 bioactivity, aiding in the differentiation between the two complications (19, 128). ICIs-induced HLH typically occurs between 3 to 15 weeks after the initiation of ICIs, with a median of 7 weeks, often observed during the third or fourth cycle of treatment (118, 129). One of the diagnostic criteria for HLH is the presence of a fever higher than 38.5°C. According to our review, patients with ICIs-induced HLH frequently exhibit hyperthermia, with peak temperatures ranging between 38.6°C and 40.5°C (115–117, 124, 130, 131).

## 4 Comparison between ICIs-related FUO with other FUO types

**Figure 2:**
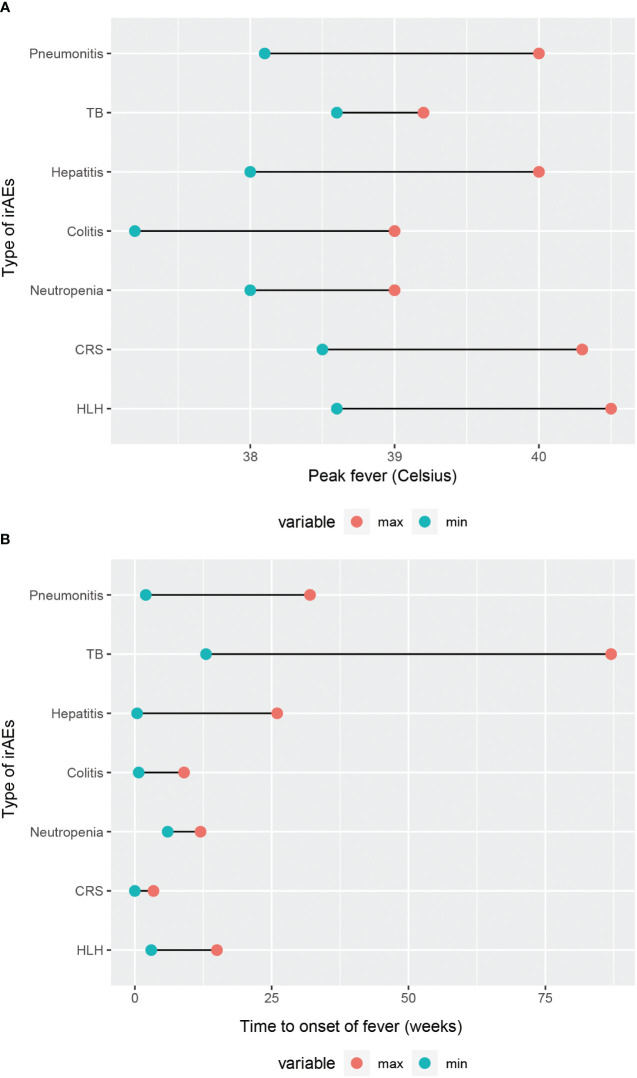
Comparison of fever condition in different types of irAEs. **(A)** Range of peak fever. **(B)** Time to onset of fever.

It is generally accepted that FUO is mainly categorized into classic FUO, nosocomial FUO, and immunodeficiency-associated FUO and others (6). In this review, we aim to summarize a novel type FUO, namely ICIs-associated FUO. First and foremost, we make comparison of the etiology, incidence, clinical presentation, diagnostic methods and fever condition in various types of ICI-associated FUO (**Table 3**). The comparison of their time to onset of fever and peak body temperature is illustrated in (**Figure 2**). It can be noted that patients with CRS and HLH tend to experience higher febrile temperatures, whereas patients with TB, IMC, and neutropenia typically present febrile temperatures below 39°C. The majority of patients develop fever symptom within 25 weeks of the initiation of ICIs, with TB being the exception, possibly because reactivation of TB is associated with the patient’s latent infection status and the nutritional and immune condition, which warrants further investigation. Notably, CRS, an acute systemic inflammatory response, has the earliest onset time of fever among all irAEs. Moreover, through the in-depth exploration of the pathogenic mechanisms, we summarized immune activation, elevation of cytokines, and infection or reactivation of pathogens as crucial features of ICI-associated FUO. Consequently, laboratory tests of cytokines and pathogens can serve as two crucial clues for diagnosis in clinical practice.

**Table 3:** Comparison between various irAEs inducing FUO after ICIs administration.

	Pneumonitis	TB	Hepatitis	Colitis	Neutropenia	CRS	HLH
Etiology associated with FUO	immune activation and cytokines secretion	immune activation and pathogen reactivation	immune activation and cytokines secretion	immune activation and cytokines secretion	immune activation and pathogen infection	immune activation and cytokines secretion	immune activation and cytokines secretion
Epidemiology	3.5% - 19%	1.7%; (1% - 6%)^**^	5%-10%	3.6%	0.94%	4.6% or lower	0.08%
More prevalent ICI model^*^	PD-1/L1	PD-1/L1	CTLA-4	CTLA-4	PD-1/L1	PD-1/L1	PD-1/L1
Incidence rate of fever	12%	NA	14%	12%	50%	All	Nearly All
Clinical presentation	dyspnea; cough; fever; chest pain	cough; fever; expectoration; weight loss	fatigue; abdominal discomfort; fever; rash; jaundice	diarrhea; abdominal discomfort; nausea; vomiting; fever	fever; weakness; pain	fever; tachycardia; headache; hypotension	fever; splenomegaly; hepatomegaly; skin rash
Laboratory tests	elevated CRP, ESR, WBC, N	acid-fast staining; sputum culture; PCR; NGS; IGRA	elevated ALT, AST, ALP, STB	elevated CRP, ESR, fecal calprotectin; anemia; stool culture	reduced N	elevated IL-6, IL-1, IFN-γ, CRP	cytopenia; hypertriglyceridemia; hypofibrinogenemia
Histologic findings	lymphocytic infiltration; granulomatous inflammation; OP	necrotic tissue layer; granulomatous inflammation	mononuclear inflammation; lobular hepatitis; granulomatous hepatitis	diffuse mucosal inflammation; acute inflammatory features	granulocyte hypoplasia; granulocyte maturation blockade, or lymphocyte infiltration	–	hemophagocytosis in bone marrow, spleen, or lymph node

ALP, alkaline phosphatase; ALT, alanine aminotransferase; AST, aspartate aminotransferase; BALF, bronchoalveolar lavage fluid; CRP, C reactive protein; CRS, cytokine release syndrome; ESR, erythrocyte sedimentation rate; HLH, hemophagocytic lymphohistiocytosis; IGRA, interferon-gamma release assay; N, neutrophil; NA, not available; NGS, next-generation sequencing; OP, organizing pneumonitis; PCR, polymerase chain reaction; STB, serum total bilirubin; TB, tuberculosis; WBC, white blood cell; d, day(s); m, month(s); w, week(s).

*confined to ICI monotherapy irrespective of combination regimen;

**1.7% is confined to lung cancer, while 1% - 6% is confined to PD-1/PD-L1 therapy.

Subsequently, we conducted a comparison between ICIs-associated FUO with classic FUO, nosocomial FUO, neutropenia-associated FUO, and HIV infection-associated FUO. The main points of comparison encompass their definitions, patient distributions, primary etiologies, key elements in history inquiry and physical examination, auxiliary tests, management, and clinical course (**Table 4**) (6, 132–134). Instead of identifying a particular cutoff as peak temperature and duration time in the definition, we emphasize that the mechanism of ICIs-associated FUO involves autoimmune activation induced by ICIs, leading to the release of EP and other fever-associated cytokines. ICIs-associated FUO occurs in both outpatients and hospitalized patients, whose potential etiologies theoretically encompass all adverse events caused by ICIs. Therefore, during history taking, attention should be paid to the patient’s medication and treatment history, as well as their cancer conditions. Targeted physical examinations and diagnostic investigations should be carried out accordingly.

**Table 4:** Comparison between ICIs-related FUO with other FUO types.

	Classic FUO	Nosocomial FUO	Neutropenia- associated FUO	HIV infection- associated FUO	ICIs-associated FUO
Definition	> 38.3°C, > 3w, > 1w clinical assessment	> 38.3°C, > 3d, no fever at admission	> 38.3°C, > 5d, despite empirical antibiotic therapy	> 38.°C, > 3w for outpatients > 3d for inpatients	fever during ICIs administration with immune activation
Distribution	community patients, outpatients, inpatients	ICU patients, non-ICU patients	inpatients, outpatients	community patients, outpatients, inpatients	inpatients, outpatients
Main Etiology	infections, NIID, cancers	nosocomial infections, postsurgical infections, drugs	infections (though only 40% - 60% of cases have pathogens identified)	HIV, HHV-8 and Mycobacterial infections; toxoplasmosis; cryptococcosis; lymphoma	pneumonitis, TB, hepatitis, colitis, neutropenia, CRS, HLH, hypophysitis, etc
History Taking	history of travel, contact, animal exposure, family, immunization, valvular heart disease	history of surgery, invasive operation, medication; medical devices implantation; anatomical structure	history of medication and primary immunodeficiency diseases; stage of chemotherapy	history of exposure, contact, travel, medication; infection stage of HIV; risk factors	history of medication (chemotherapy, immunotherapy, targeted therapy); radiotherapy; cancer conditions
Physical Examination	fundi, oropharynx, temporal artery, heart, abdomen, lymph nodes, spleen, joints, skin, nails, genitalia, rectum, prostate, deep veins	wound, drainage tube, implanted medical devices, sinus tract, urine	skin folds, venipuncture site, lungs, perianal region	oral cavity, nasal sinuses, skin, lymph nodes, eyes, lungs, perianal region	skin, liver, lungs, abdomen, lymph nodes, thyroid, heart, nervous system
Auxiliary Tests	based on diagnostic clues	imaging tests; bacterial culture	thoracic imaging tests; bacterial culture	CBC; serological tests; thoracic and cranial imaging tests; fecal tests; lung, liver, and marrow biopsy; bacterial culture	cytokines level; pathogen tests; CBC; CRP; ESR; blood biochemistry; thoracic and abdominal imaging tests;
Management	observation; recording of body temp; conducting auxiliary tests; avoiding empirical medication	based on patients’ condition	antibacterial medication	antiviral and antibacterial medication; vaccination	suspend ICIs; corticosteroid; anti-cytokines antibody; antimicrobial medication;
Course	several w - m	several d -w	several d - 1 w	several w - m	several w - m

CBC, complete blood count; NIID, non-infectious inflammatory and autoimmune diseases; d, day(s); m, month(s); temp, temperature; w, week(s).

## 5 Discussion

It is common for cancer patients to experience fever triggered by the tumor, infection, and various treatment patterns. Drug-induced fever in cancer patients has grabbed sufficient attention, as relevant guidelines have been developed (37). It is of vital importance to clarify the causes and mechanisms of fever. The mechanisms of irAEs include augmented T cells’ response to normal and tumor tissues, elevated levels of cytokines, increased levels of autoantibodies, and enhanced complement-mediated inflammation (135). However, while this might account for the occurrence of Haem irAEs induced by ICIs, it falls short of demonstrating the specificity of adverse events occurring in individual solid organs. This review delves into the immunological mechanisms of pneumonitis, TB, hepatitis, and colitis caused by ICIs separately and finds that they all include abnormal changes in cytokines’ level. Therefore, we propose that the elevation of multiple cytokines and immune activation are a shared characteristic among them.

In addition to aforementioned compilations, there is a potential of other system disorders, such as encephalitis and meningitis, belonging to central nervous system diseases, presenting with fever (136). Because these adverse effects occur relatively less, they are inadequately documented and the potential mechanism still waits for further exploration (137, 138). Notably, they can result in poor prognosis, even fatality, which should not be overlooked in clinical practice (139).

It is noteworthy that the incidence of irAEs, the onset time of them, and the severity vary for different types of cancers and drugs. In general, the incidence and severity of adverse events at all grades are higher for anti-CTLA-4 inhibitors (135, 140). However, concerning fever solely, there seems to be a lack of extensive statistic on the incidence, onset time, and severity of fever among various irAEs caused by anti-PD-1/PD-L1 and anti-CTLA-4 inhibitors. In the section of IMH, we mention that fever is more prevalent with anti-CTLA-4 inhibitors than with anti-PD-1/PD-L1 therapy (66), but there is still a lack of statistically rigorous analyses, leaving room for future exploration. As biomarkers are a hot spot in medical research at present, exploring whether we can predict the occurrence and severity of fever in ICIs-treated patients by assessing baseline and post-treatment levels and alterations in multiple cytokines represents an avenue for future efforts. Additionally, exploring the potential for designing preventive and therapeutic measures based on these biomarkers is an area worth considering.

Consistent with the traditional view, we regard fever as an adverse symptom following ICIs treatment. In other words, we focus on the process by which ICIs induce fever, rather than the effect that fever exerts on ICIs treatment. It is reported that fever regulates the tumor immune microenvironment and enhances the immune response through heat shock protein (141). Considering that fever can promote damage to tumor DNA and induce immunogenic cell death, fever may play a positive role in suppressing tumor growth, activating the immune system and enhancing the efficacy of ICIs (138). This perspective is advantageous for a dialectical consideration of the physiological significance of ICIs-associated FUO.

## Author contributions

XT: Writing – review & editing, Writing – original draft. TZ: Writing – review & editing, Writing – original draft. XD: Writing – review & editing, Supervision, Formal Analysis. DX: Writing – review & editing, Supervision, Methodology, Conceptualization.
